# Enhancing the Mechanical Performance of Dual-Phase Steel Through Multi-Axis Compression and Inter-Critical Annealing

**DOI:** 10.3390/ma18133139

**Published:** 2025-07-02

**Authors:** Pooja Dwivedi, Aditya Kumar Padap, Sachin Maheshwari, Faseeulla Khan Mohammad, Mohammed E. Ali Mohsin, SK Safdar Hossain, Hussain Altammar, Arshad Noor Siddiquee

**Affiliations:** 1 Department of Mechanical Engineering, Inderprastha Engineering College, Ghaziabad 201010, Uttar Pradesh, India; 2 Department of Mechanical Engineering, Bundelkhand Institute of Engineering and Technology, Jhansi 284128, Uttar Pradesh, India; padap@rediffmail.com; 3 Department of Mechanical Engineering, Netaji Subhas University of Technology (Formerly Netaji Subhas Institute of Technology), New Delhi 110078, India; ssaacchhiinn@gmail.com; 4 Department of Mechanical Engineering, College of Engineering, King Faisal University, Al-Ahsa 31982, Saudi Arabia; haltammar@kfu.edu.sa; 5 Department of Chemical Engineering, College of Engineering, King Faisal University, Al-Ahsa 31982, Saudi Arabia; maa.ali@kfu.edu.sa (M.E.A.M.); snooruddin@kfu.edu.sa (S.S.H.); 6 Department of Mechanical Engineering, Jamia Millia Islamia, New Delhi 110025, India; arshadnsiddiqui@gmail.com

**Keywords:** dual-phase steel, inter-critical annealing process, multi-axis compression, microstructure, mechanical properties, wear test

## Abstract

This study examines the microstructural evolution, mechanical properties, and wear behavior of medium-carbon dual-phase steel (AISI 1040) processed via Multi-Axis Compression (MAC). The DP steel was produced through inter-critical annealing at 745 °C, followed by MAC at 500 °C, resulting in a refined grain microstructure. Optical micrographs confirmed the presence of ferrite and martensite phases after annealing, with significant grain refinement observed following MAC. The average grain size decreased from 66 ± 4 μm to 18 ± 1 μm after nine MAC passes. Mechanical testing revealed substantial improvements in hardness (from 145 ± 9 HV to 298 ± 18 HV) and ultimate tensile strength (from 557 ± 33 MPa to 738 ± 44 MPa), attributed to strain hardening and the Hall–Petch effect. Fractographic analysis revealed a ductile failure mode in the annealed sample, while DP0 and DP9 exhibited a mixed fracture mode. Both DP0 and DP9 samples demonstrated superior wear resistance compared to the annealed sample. However, the DP9 sample exhibited slightly lower wear resistance than DP0, likely due to the fragmentation of martensite induced by high accumulated strain, which could act as crack initiation sites during sliding wear. Furthermore, wear resistance was significantly enhanced due to the combined effects of the DP structure and Severe Plastic Deformation (SPD). These findings highlight the potential of MAC processing for developing high-performance steels suitable for lightweight automotive applications.

## 1. Introduction

The automotive sector today is undergoing rapid transformation, driven by the growing adoption of electric vehicles and fierce competition to improve fuel efficiency. These trends have pushed manufacturers to develop lighter, safer, and more fuel-efficient vehicles without compromising performance. Meeting these demands requires innovative materials that can balance seemingly opposing properties—such as high strength and ductility. Advanced High-Strength Steels (AHSSs), particularly the dual-phase (DP) steels, have emerged as a key solution because of their strength–ductility combination, offering the durability, safety, efficiency, and cost-effectiveness the industry needs [1].

Among the latest generation of AHSSs, DP steel, along with Transformation-Induced Plasticity (TRIP) and Twinning-Induced Plasticity (TWIP) steels, are widely used in automotive manufacturing [2]. DP steel’s unique microstructure—hard martensite islands embedded in a softer ferrite matrix—gives it an ideal blend of strength (from martensite) and ductility (from ferrite) [3]. This structure is typically achieved through inter-critical annealing, where the steel is heated into the ferrite (α) + austenite (γ) phase region and then rapidly cooled, transforming austenite into martensite [4,5]. Due to its dual-phase microstructure, the DP steel exhibits better strength–ductility synergy, making it suitable for structural automotive parts like wheel discs, body panels, and bumper reinforcement beams [6,7]. However, many industrial applications—particularly sheet metal forming—require materials that can withstand large plastic deformation while maintaining high strength, a rare combination due to the inherent trade-off between these properties [8]. Research has extensively explored how martensite influences DP steel’s mechanical behavior, confirming that inter-critical annealing optimizes strength and ductility [9]. While increasing martensite content boosts strength, it often reduces ductility. Grain refinement offers a promising way to enhance strength without severely sacrificing ductility [10].

One effective way to refine grains is through Severe Plastic Deformation (SPD) techniques, which apply intense strain to bulk materials, reducing grain size to submicron or even nano-meter scales [11].The resulting ultrafine-grained (UFG) microstructures can be more than twice as strong as their coarse-grained counterparts [12]. Common SPD methods include Accumulative Roll Bonding (ARB), Equal Channel Angular Pressing (ECAP), High-Pressure Torsion (HPT), and Multi-Axis Compression (MAC). Among these, MAC is particularly attractive for its simplicity, cost-effectiveness, and ability to impose large strains without altering the sample’s overall shape. It can produce UFG or nanocrystalline structures and is compatible with standard industrial forging equipment [13]. In MAC processing, the material undergoes multi-stage forging, with the compression axis rotated in each stage—typically along three orthogonal directions. Padap et al. showed that multi-axial forging of AISI 1060 steel significantly improves mechanical properties through grain refinement [14,15].

AISI 1040 steel is a widely utilized candidate for industrial applications, namely automobile, shipbuilding, and aerospace, owing to its balanced mechanical properties. Although the development of material is at its peak, carbon steels remain the most extensively used material owing to their low cost, availability, and excellent machinability [16]. Kayali et al. used this grade of steel to analyze the wear behavior after diffusion annealing and found that wear resistance was enhanced [17]. Heat-treated AISI 1040 steel was used for the assessment of surface characterization using a magnetic non-destructive technique [18]. Similarly, spheroidization and related heat treatments was performed on AISI 1040 steel, and its machinability studied by varying room temperature [19].

While DP steels and MAC processing have been studied separately, there is limited research on how MAC affects medium-carbon DP steels. This study aims to bridge that gap by enhancing the mechanical and wear properties of medium-carbon steel (AISI 1040) through inter-critical annealing followed by MAC, unlocking its potential for advanced engineering applications. Therefore, the following research sequence was followed in this work: (i) AISI 1040 steel was homogenized, (ii) the homogenized steel was inter-critically annealed to form dual-phase steel, and (iii) Multi-Axis Compression was performed to enhance the mechanical properties. The microstructural and mechanical aspects during these steps were investigated to reveal the structure–property relationships.

## 2. Materials and Methods

Medium-carbon steel (AISI 1040, Make: Alfa Aesar, Haverhill, MA, USA)with the chemical composition listed in Table 1 was selected for this investigation. Prismatic specimens with dimensions of 20 mm × 16.33 mm × 13.33 mm were machined, maintaining a dimensional ratio of 1.5:1.22:1.0 to preserve geometric stability during the MAC process. Prior to MAC and inter-critical annealing, the specimens were homogenized by annealing at 920 °C for 50 min in a muffle furnace (Nabertherm GmbH, Lilienthal, Germany), followed by furnace cooling. This step eliminated the effects of prior work hardening and ensured a microstructural uniformity.

**Figure 1:**
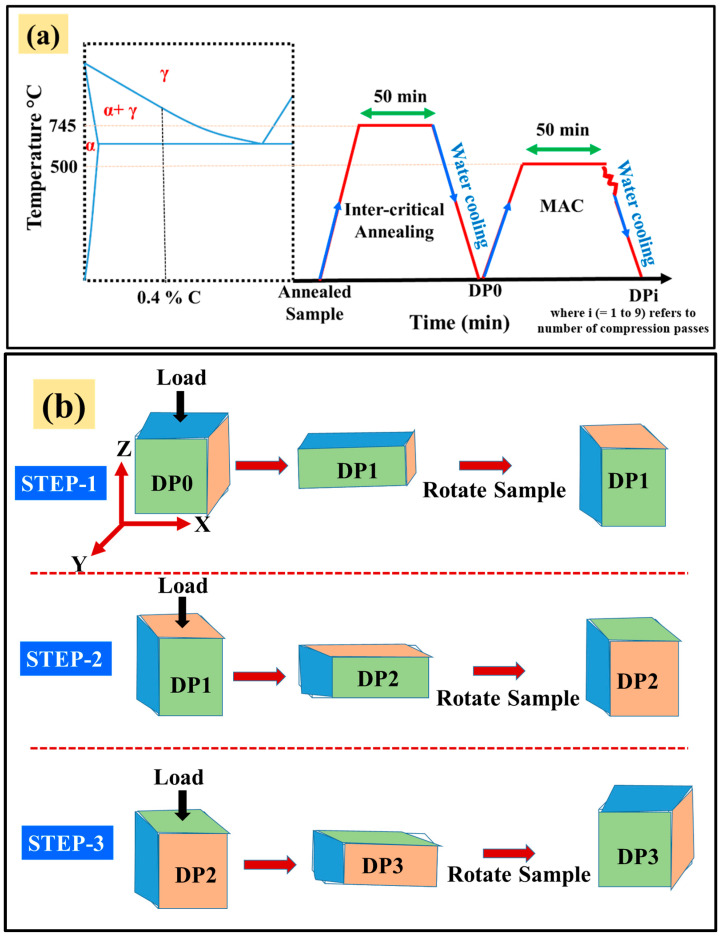
(**a**) Schematic of the phase diagram showing inter-critical annealing and the MAC process (α-ferrite, γ-Austenite) and (**b**) schematic of sequence of MAC operations along X-, Y-, and Z-directions.

The homogenized samples underwent inter-critical annealing at 745 °C for 50 min, followed by water quenching to produce DP steel. These initial specimens were labeled DP0. The DP0 steel was then processed via Multi-Axis Compression (MAC) at 500 °C, with a compressive strain of 0.4 per pass. Figure 1a illustrates the detailed sequence of inter-critical annealing and MAC processing. During the first MAC stage, the DP steel samples were heated to 500 °C, held for 50 min, and compressed along the X-direction before water quenching. This procedure was repeated sequentially along the Y- and Z-directions for subsequent passes. After each pass, the samples were labeled as DP1, DP2, DP3…DPi, where i refers to the number of compression passes. A complete MAC cycle consisted of three compressive passes along orthogonal directions (X, Y, and Z). In each pass, compression was applied along the specimen’s longest axis with a true strain of 0.4, as shown in Figure 1b. Thus, the cumulative plastic strain after each pass increases as 0.4, 0.8, 1.2, 1.6 for the DP1, DP2, DP3 samples, respectively, and the total logarithmic plastic strain of 3.6 prevails for the DP9 sample. To maintain dimensional integrity and ensure isotropic deformation, we adhered to the principles of volume conservation and directional consistency. Graphite powder (Loba Chemie Pvt. Ltd., Mumbai, India) was used as a lubricant to minimize friction and prevent surface cracking during compression [8]. The images of steel samples at various thermomechanical steps are shown in Figure S2 of the supplementary file. It should be noted that A1 and A3 temperatures for the studied material are 727 °C and 912 °C, respectively. Whereas A3 is not a fixed temperature; instead it is the upper critical temperature of steel in the Fe-C phase diagram. It varies from 912 °C for 0 wt.% C carbon (pure iron α-Ferrite) to 0.76 wt.% C steel where its value is 727 °C. At 0.4 wt.% C, the upper critical temperature is about 780 °C, hence a suitable value of 745 °C is taken between 727 °C and 780 °C. The temperature of 500 °C for the MAC processing was selected as per the authors’ previous research on MAC [15]. Also, since the 0.4 wt.% C steel is hard (i.e., hardness ~145 HV), performing MAC at room temperature may cause cracking. Therefore, warm MAC (heating below recrystallization temperature) was performed, which helps uniform deformation without cracking.

Microstructural analysis was performed using optical microscopy (OM; Olympus BX53M, Olympus Corporation, Tokyo, Japan) and scanning electron microscopy (SEM; ZEISS EVO18, Carl Zeiss AG, Oberkochen, Germany). Samples were prepared via standard metallographic polishing, involving sequential grinding with SiC papers (Struers A/S, Ballerup, Denmark) of grades #100 to #2000 and subsequent polishing with alumina suspension (Buehler, Lake Bluff, IL, USA) on a polishing cloth. They were then etched in a 2% Nital solution (2 mL HNO_3_ + 98 mL C_2_H_5_OH from Merck KGaA, Darmstadt, Germany). Since the material exhibited a two-phase microstructure consisting of ferritic matrix and embedded pearlite or martensite phases, two approaches were followed for the measurement of their respective sizes. For the ferritic matrix, grain size measurements were conducted using the linear intercept method which can be simplified as mentioned in Equation (1) below:

(1) D=Ltn,

where *D* is the average grain size, $L_{t}$ is the length of the intercepting lines, and n is the number of interceptions with the ferritic grain boundaries. The intercepting lines were made at an angle of 0°, 45°, 90°, and 135° to account for the change in grain size with direction. On the other hand, the size of the pearlite and martensitic phases was measured by calculating the average of the individually measured diameters of these embedded phases in different directions as per their aspect ratio.

Vickers hardness testing was carried out as per the ASTM E92 standards [20], on metallographically prepared specimens using a 5 kgf load and a 10 s dwell time. For each sample, the average Vickers hardness measurement was calculated from 5 indentations. The distance between indentations was kept significantly larger to avoid any errors resulting from local deformation at neighboring indents. The apparatus for Vickers hardness measurements is shown in supplementary Figure S1a.

Miniature tensile specimens having a dog-bone shape with dimensions of 20 mm total length, 8 mm gauge length, and a thickness and width of 0.75 mm and 1.5 mm, respectively, were extracted using wire electrical discharge machining (EDM). The specimen dimensions were selected as per the requirements defined by Hyde et al. [21], and tests were conducted on a universal testing machine at a strain rate of 2 × 10^−3^ s^−1^ at room temperature to evaluate mechanical performance.

Dry sliding wear tests were performed as per the ASTM G99 standards [22], using a pin-on-disc tribometer (DUCOM TR-20LE, DUCOM Instruments Pvt. Ltd., Bengaluru, India), with EN-31 hardened steel as the counter face disc, as shown in supplementary Figure S1b. Tests were conducted at a constant sliding speed of 1 m/s under three different normal loads: 9.8 N, 19.6 N, and 29.4 N. Each test spanned a total duration of 30 min, segmented into three equal intervals of 10 min. The volume loss was calculated from weight loss measurements taken at each interval using a precision balance. Post-wear surface morphology was examined via SEM to identify the dominant wear mechanisms.

## 3. Results and Discussions

### 3.1. Microstructural Evolution

**Figure 2:**
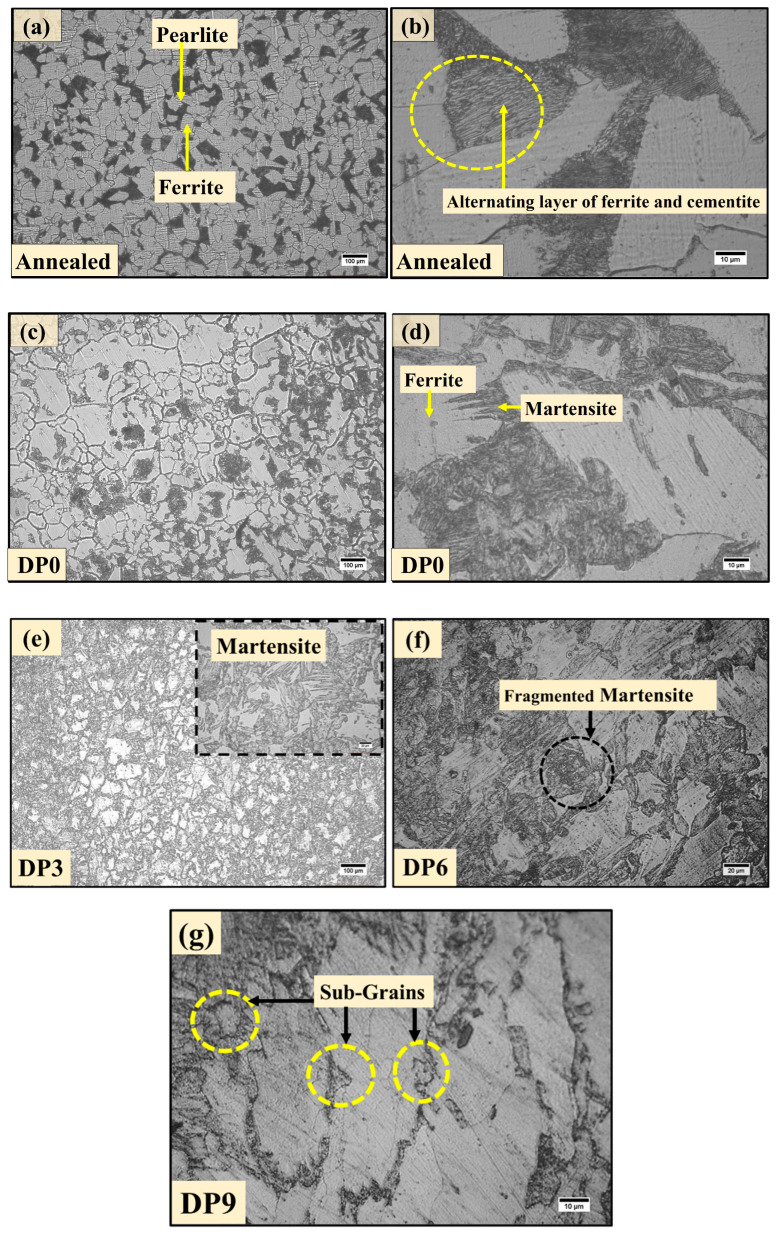
Microstructures of the annealed, inter-critical annealing, and MAC-processed samples: (**a**) annealed, (**b**) magnified view of (**a**), (**c**) DP0 (inter-critical annealing alone), (**d**) magnified view of (**c**), MAC microstructures: (**e**) DP3, (**f**) DP6, and (**g**) DP9 samples.

The initial microstructure of the steel sample annealed at 920 °C for 50 min is presented in Figure 2a. The microstructure consists of distinct white and dark regions, corresponding to ferrite and pearlite phases, respectively. A higher magnification view (Figure 2b) reveals that the pearlite comprises alternating lamellae of α-ferrite (light phase) and cementite (dark phase). These lamellae are organized into pearlite colonies, within which the lamellae maintain a nearly parallel alignment, although their orientation varies between colonies [11] Quantitative image analysis indicates that the initial microstructure contains approximately 66 vol.% ferrite, with an average grain size of 66 ± 4 μm, and about 34 vol.% pearlite having an average diameter of 36 μm.

Following inter-critical annealing at 745 °C for 50 min and subsequent water quenching, partial transformation of ferrite to austenite occurs, which is subsequently transformed into martensite upon quenching. The resulting DP0 microstructure, shown in Figure 2c, consists of ferrite and approximately 39 vol.% martensite. The martensite appears as dark islands distributed within the bright ferrite matrix. At higher magnification in Figure 2d, the martensite exhibits a needle-like morphology embedded in the ferrite phase.

The evolution of microstructure under MAC is depicted in Figure 2e,f for the DP3, DP6, and DP9 conditions, corresponding to three, six, and nine passes of deformation, respectively. The area fraction of martensite increased from ~39% in the DP0 sample, i.e., after water quenching, which increased to ~44% in DP3 sample and to ~48% in the DP6 and DP9 samples after MAC. A low magnification image of DP9 used for estimating the area fraction is shown as supplementary Figure S3. The increase in the fraction of martensite with MAC is in accordance with the increase in hardness and decrease in ductility, as discussed in Section 3.2 and observed in previous works [23]. The progressive increase in the hardness correlates with the increase in concentration of the martensite and balance being ferrite. It is to be noted that the ferrite phase is characteristically soft and ductile, and consequently bears significant deformation, and the DP steel continues to contribute sufficient ductility along with enhanced strength and hardness. After three passes (DP3), the ferrite grains exhibit deformation-induced substructures, which are elongated grains, as seen in Figure 2e. This is indicative of early-stage sub-grain formation and strain accumulation. Concurrently, the martensite islands retain their morphology but begin to exhibit localized deformation. With an increasing number of passes (DP6), the martensite phase becomes increasingly fragmented due to accumulated local stress and is redistributed more uniformly within the ferritic matrix (Figure 2f). This fragmentation facilitates strain accommodation and contributes to a more homogeneous deformation response across the DP microstructure. Additionally, the ferrite grains exhibit progressive refinement via continuous dynamic recrystallization (CDRX) mechanisms [15,23,24,25], as indicated by the evolution of equiaxed sub-grains. After nine passes of MAC (DP9), the microstructure shows a high density of refined substructures and equiaxed grains with a near-uniform distribution of fragmented martensite and refined ferrite as shown in Figure 2g. The size of sub-grains for the DP9 sample in Figure 2g was measured as 8 ± 1.1 μm. This transformation is primarily driven by the imposed SPD under multi-axial loading. The periodical change in compression axis with each MAC pass introduces non-monotonic, multi-directional strain paths and periodic stress reversals, which accelerate microstructural refinement through fragmentation of martensitic features and enhanced grain subdivision in ferrite [26,27]. The cumulative effect of these mechanisms results in a significant reduction in average grain size from 66 ± 4 μm in the undeformed condition to 18 ± 1 μm after nine passes of MAC. This highlights the efficacy of MAC in promoting grain refinement and microstructural homogenization in DP steels which enhances the mechanical properties.

### 3.2. Mechanical Properties

**Figure 3:**
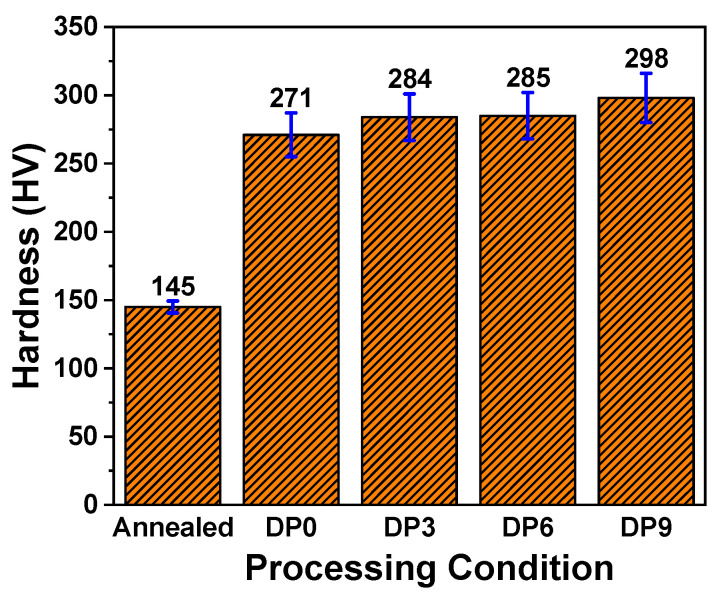
Hardness of the various processed samples.

The microstructure evolution induced by MAC directly influences the mechanical properties of the studied material. The hardness measurements of the processed material are presented as a function of MAC passes in Figure 3. The annealed sample exhibited an average Vickers hardness of 145 ± 9 HV, while the hardness increased markedly in the DP0 sample and continued to rise with additional MAC passes, reaching 298 ± 18 HV in the DP9 condition. The initial increase in hardness from the annealed to DP0 state is approximately twice which is attributed to the formation of a dual-phase ferrite–martensite microstructure with enhancement of the martensite volume fraction by approximately 13% (Figure 2c), which indicates that hard martensitic phase significantly enhances resistance to plastic deformation.

With progressive MAC passes, the hardness increase is further driven by two key mechanisms: (i) grain refinement, which raises grain boundary density through formation of fine grains and limits dislocation motion, consistent with the Hall–Petch relationship [28], i.e.,

$$\sigma_{y}=\sigma_{0\;}+kd^{1/2}$$

where $\sigma_{y}$ is the yield stress, $\sigma_{0\;}$ is the friction stress against the dislocation movement, *k* is the Hall–Petch coefficient, and *d* is the grain diameter; (ii) strain hardening, arising from the accumulation of dislocations and formation of substructures under SPD. In this strengthening process, the ferrite phase significantly contributes through dislocations which are generated during processing and dislocation motion is hindered by grain boundaries, second-phase particles, and dislocations themselves. This hindrance is the resistance to plastic deformation which indeed strengthen the processed material [29].

The high temperature driven CDRX supports recovery and periodic stress reversals, enhancing sub-grain formation and further refining the microstructure (Figure 2g) [30,31].

**Figure 4:**
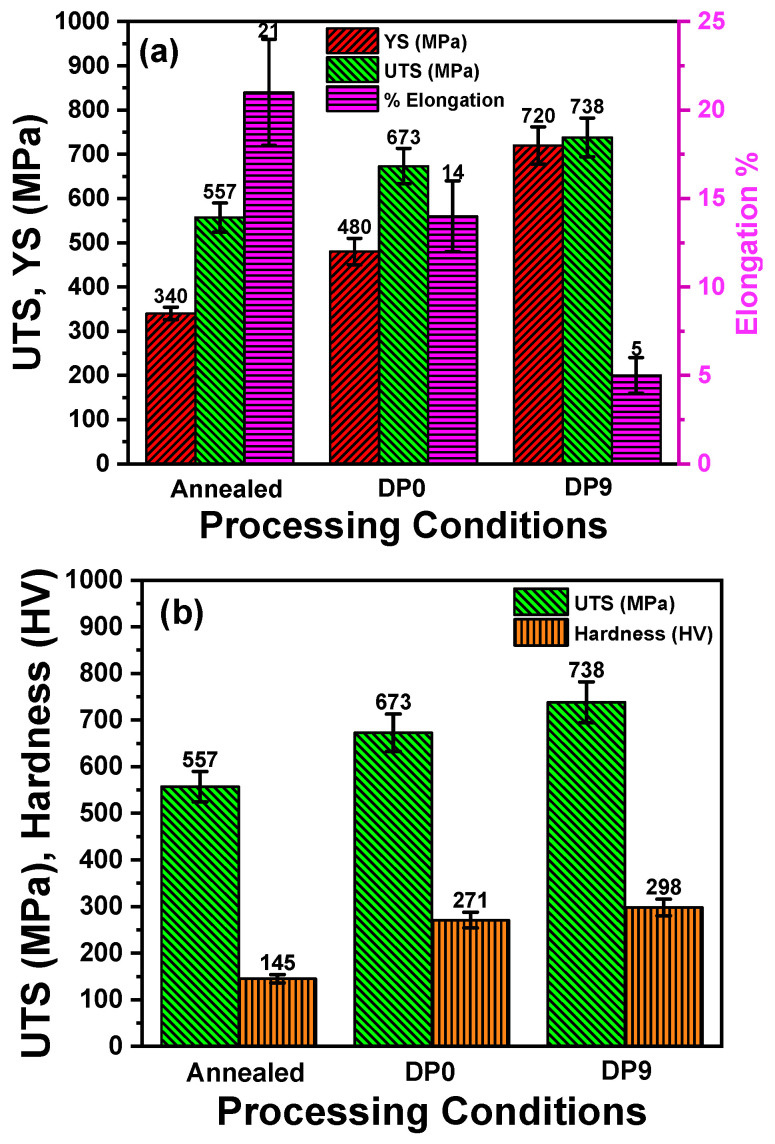
(**a**) YS, UTS, and elongation for annealed and MAC-processed samples and (**b**) comparison of evolution in UTS and hardness values for annealed, DP0, and DP9 samples.

Figure 4a presents the bar graph for true stress–strain curves and ductility or the annealed, DP0, and DP9 samples. The yield strength (YS) of the sample increases from 340 ± 14 MPa in the annealed condition to 720 ± 42 MPa in DP9 sample. Similarly, the ultimate tensile strength (UTS) increased from 557 ± 33 MPa in the annealed condition to 673 ± 40 MPa in DP0, and further to 738 ± 44 MPa after nine MAC passes. This progressive improvement is attributed to a combination of phase transformation (formation of martensite), strain-induced dislocation interactions, and grain refinement [32,33]. The transformation from a coarse-grained ferrite–pearlite microstructure (Figure 2a) to a fine-grained ferrite–martensite structure (Figure 2g), coupled with strain-induced dislocation interactions, provides dual strengthening effects: (i) grain boundary strengthening and (ii) dislocation forest hardening. As dislocations accumulate and interact, their mobility is increasingly restricted, resulting in elevated flow stress during deformation. The ferrite phase is the major driver for the dislocation-assisted strengthening during MAC and simultaneously accommodating the strain, though of course, the former continuously increased at the cost of reduction in the values of the latter. Additionally, the homogeneous dispersion of fragmented martensite improves the compatibility between phases, reducing localized strain incompatibilities and further stabilizing the mechanical response. These mechanisms collectively restrict dislocation motion and delay the onset of plastic instability, thereby enhancing strength [34,35]. The increase in dislocation interactions discussed above understandably occurs due to the increase in dislocation density. Upon multi-axial forging, the dislocation density of interstitial free steel increased from ~4.2 × 10^13^ m^−2^ in the undeformed material to ~1.1 × 10^15^ m^−2^ after the first cycle and to ~1.3 × 10^15^ m^−2^ after the second cycle [36]. Notably, this increase occurs by two orders of magnitude in the first cycle. This was also observed in another study, where the initial dislocation density of ~1 × 10^12^ m^−2^ increased to ~2.2 × 10^14^ m^−2^ after the first cycle [37].

**Figure 5:**
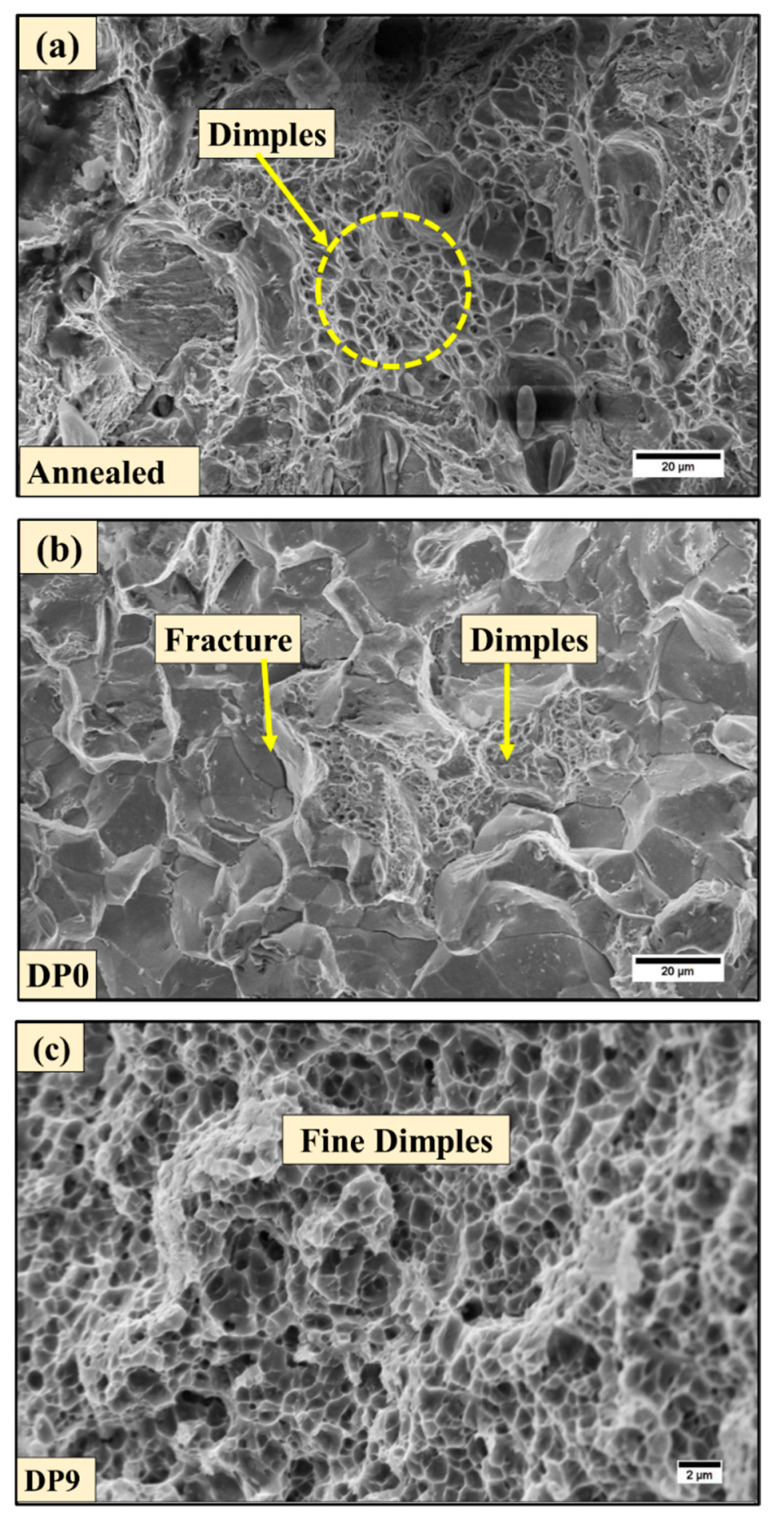
Fracture surface morphology of the different processed samples: (**a**) annealed (**b**) DP0, and (**c**) DP9 samples.

It must be noted that a significant reduction in ductility was observed as the number of passes increased from 0 to 9. The ductility of the DP0 steel was ~14 ± 1% compared to 5 ± 0.5% after nine passes of MAC. Since grain boundaries act as barriers to dislocation motion, the reduction in grain size upon nine successive MAC passes decreases the mean free path of the dislocations. The microstructure consisting of a smaller grain size thus has much less capacity to accommodate the plastic strain. Furthermore, the progressive fragmentation of martensite particles leads to the pinning of dislocations, which further restricts the dislocation movement in the slip systems of BCC ferrite. Hence, the significant increase in strength is compensated by a reduction in ductility. This phenomenon is commonly observed in nearly all metallic materials where the secondary phase increases the strength but at the expense of ductility [38]. A comparison of evolution in UTS and hardness values for annealed, DP0, and DP9 samples is also shown in Figure 5b. As expected, both the UTS and hardness values show similar increasing trends. Therefore, the enhancement in mechanical properties occurs synergistically at both the local and bulk scales.

Fractographic analysis (Figure 5) reveals the corresponding failure mechanisms and correlates closely with the evolving microstructure. The annealed sample exhibits ductile fracture, with large and deep dimples indicating significant plasticity and void coalescence (Figure 5a). The sample DP0 in Figure 5b can be observed to exhibit numerous flat facet-like features across the fracture surface. The size of these facets is comparable to the grain size observed from the microstructure of the DP0 sample in Figure 2c, which indicates the presence of intergranular failure. Additionally, several smaller dimples can also be observed in Figure 5b. Therefore, it can be inferred that the fracture mode transitions to a mixed ductile–brittle mode. This transition in failure mode can be attributed to the presence of martensite, which contributes to brittle cleavage facets and intergranular fracture, while ferrite retains ductility, producing the smaller dimples. In the DP9 sample, the fracture surface reveals a refined dimple structure with occasional brittle regions (Figure 5c). The reduced dimple size is indicative of significant grain refinement and higher strength. This shift is attributed to the accumulation of microstructural stress concentrations resulting from martensite fragmentation and possible carbide precipitation at interfaces. Moreover, the retained austenite stabilized under MAC-induced strain and suppressed carbon diffusion may transform under stress and contribute to crack blunting and localized ductility, further influencing the fracture morphology [39,40].

### 3.3. Wear Behavior and Mechanism

**Figure 6:**
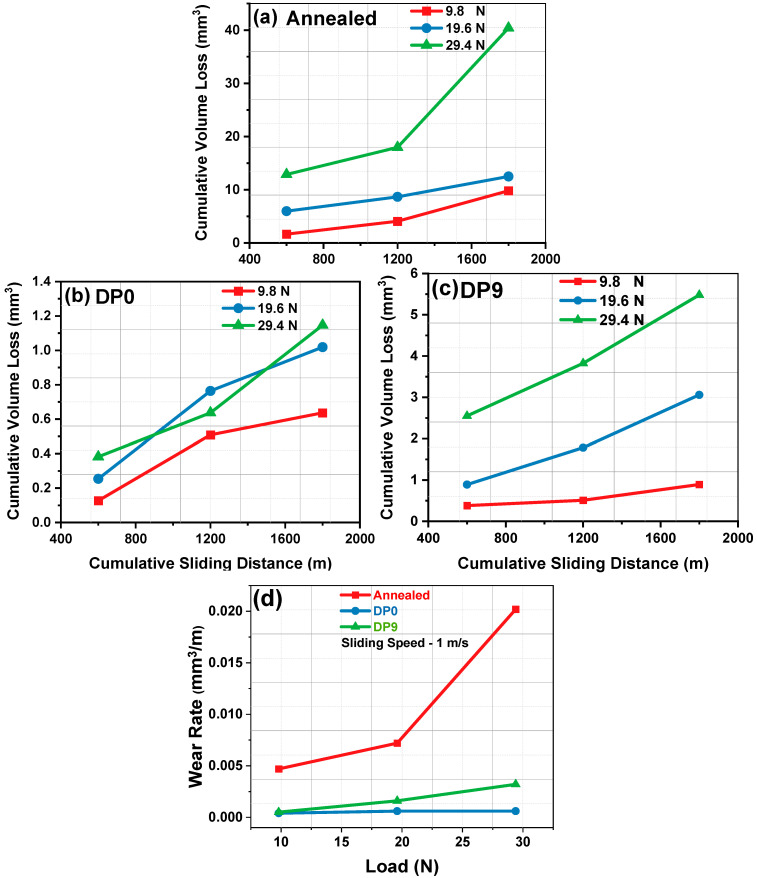
Cumulative volume loss vs sliding distance of the samples processed at different conditions: (**a**) annealed, (**b**) DP0, (**c**) DP9, and (**d**) wear rate vs load of the different samples.

The dry sliding wear performance of the annealed, DP0, and DP9 samples was evaluated at room temperature at a constant sliding speed of 1 m/s under various normal loads (9.8, 19.6, and 29.4 N) and sliding distances (600, 1200, and 1800 m). As shown in Figure 6, all samples exhibit an increase in wear volume with increasing load and sliding distance, consistent with Archard’s law [41]. This trend was observed across all samples, with maximum volume loss occurring at 29.4 N and minimum at 9.8 N. However, the annealed sample consistently exhibits the highest wear volume due to its coarse ferrite–pearlite microstructure (Figure 2a) and low bulk hardness (Figure 3). The DP0 sample, with a dual-phase ferrite–martensite structure (Figure 2c), shows reduced wear volume owing to higher hardness (Figure 3) and improved resistance to plastic deformation. The introduction of martensite into the ferritic matrix enhances load-bearing capability and reduces surface ploughing during sliding contact. However, the most refined structure (DP9) does not always show the lowest wear volume, especially under high-load conditions. Despite having the highest hardness, DP9 exhibits marginally greater wear than DP0 at 29.4 N. This can be attributed to martensite fragmentation and strain localization induced by high cumulative strain during MAC. Fragmented martensite particles may act as preferential crack initiation sites, promoting micro-chipping and delamination under severe contact stresses.

The improved wear resistance in both DP0 and DP9 is primarily governed by the refined microstructure and increased hardness, which reduces plastic flow and surface damage. Despite its higher UTS, improved by approximately 8.8% compared to DP0 and 24% compared to the annealed sample, the DP9 sample showed a slight reduction in wear resistance relative to DP0 at the highest load. This can be attributed to the extensive strain (~3.6) induced by nine MAC passes, which led to martensite fragmentation. This fragmented martensite can serve as potential crack initiation sites, contributing to increased volume loss under severe loading. However, wear resistance is also influenced by the distribution, morphology, and integrity of phases [42]. In DP9, the excessive fragmentation of martensite weakens phase continuity and introduces heterogeneity at the microscale, reducing the material’s ability to uniformly accommodate sliding-induced stresses. Figure 6d shows that although wear rate increases with load for all samples, DP0 and DP9 maintain significantly lower wear rates than the annealed condition. This improvement is attributed to enhanced deformation resistance and phase (martensite) hardness. Nevertheless, at higher loads, increased contact area and frictional heating facilitate interfacial adhesion and mechanical interlocking between asperities. These conditions can lead to unstable wear mechanisms such as micro-cutting, adhesion, or oxidative wear. In DP9, the higher strain energy stored during MAC may also increase susceptibility to localized deformation and damage accumulation under repeated sliding cycles.

**Figure 7:**
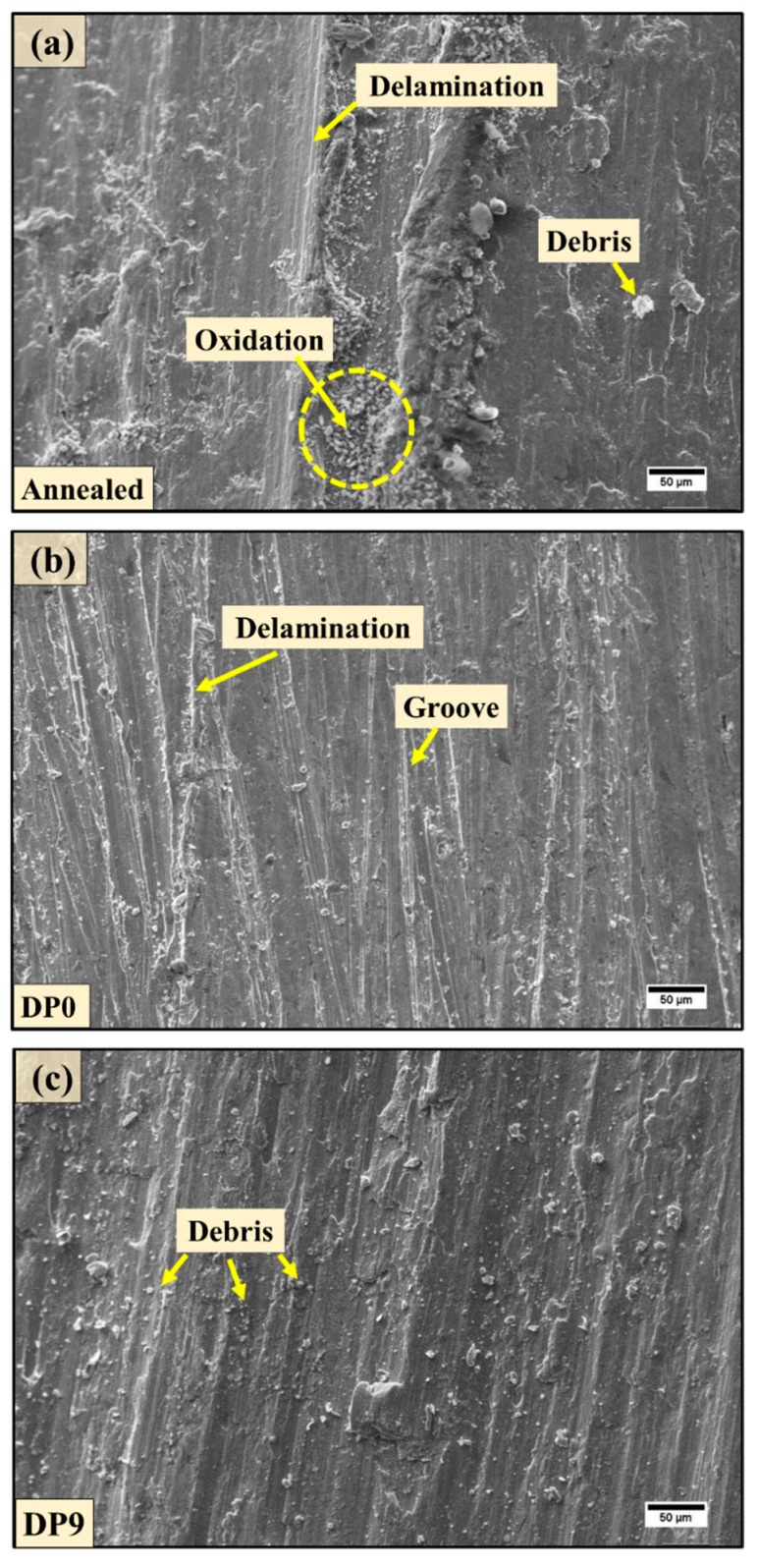
Surface morphology of worn-out samples at different processing conditions: (**a**) annealed, (**b**) DP0, and (**c**) DP9 samples.

To further support these observations, the worn surfaces were analyzed under SEM at 29.4 N (Figure 7). The annealed sample (Figure 7a) exhibited severe delamination, prominent grooves parallel to the sliding direction, and significant wear debris, indicative of dominant abrasive and delamination wear. In contrast, DP0 and DP9 samples (Figure 7b,c) displayed fewer grooves and reduced wear debris due to the dual-phase microstructure and grain refinement resulting from SPD. Nevertheless, DP9 showed more wear debris than DP0, corroborating the hypothesis of martensite fragmentation-induced wear. This also corroborates well with the lower resistance to wear as discussed. In summary, the wear mechanism across all samples is predominantly abrasive, as evidenced by the parallel grooves and surface debris [43]. While the MAC-processed DP9 sample demonstrates improved hardness and tensile strength, its slightly reduced wear resistance relative to DP0 is attributed to the strain-induced fragmentation of martensite, emphasizing the trade-off between strength enhancement and wear resistance under severe deformation. It is important to discuss the wear resistance from an application-driven perspective. The enhancement in wear resistance achieved in this study is intended for components such as brake pads, clutch plates, camshafts, and agricultural tools, mineral processing and mining equipment where steel parts slide against other materials under dry contact conditions with surface contact. These applications demand materials with not only high strength but also enhanced surface durability, which has been tuned in this study using thermomechanical processing [44,45].

## 4. Conclusions

In the present experimental study, AISI 1040 steel was used for analysis, wherein microstructural evolution, mechanical properties, and wear behavior of DP steel processed through MAC were analyzed. Although this grade of steel has been extensively studied, MAC is a relatively recent technique employed for grain refinement, which enhances strength and significantly influences tribological performance. Furthermore, inter-critical heat treatment transforms the steel into a DP structure, making it more suitable for automotive applications. The combined application of both techniques results in a material with high strength, refined microstructure, and improved tribological properties. The key findings are as follows:

1. The microstructural analysis confirmed progressive grain refinement due to the imposed strain during MAC. The formation of fine substructures in ferrite was observed after six MAC passes (DP6), which further evolved into well-defined fine grains by DP9.
2. The average grain size was reduced from 66 ± 4 μm in the annealed condition to approximately 18 ± 1 μm in the DP9 sample, attributed to the cumulative strain introduced during MAC.
3. The hardness of the DP9 sample increased by approximately 87% compared to the annealed sample and 10% compared to the DP0 sample. This improvement is primarily due to strain hardening and grain refinement governed by the Hall–Petch relationship.
4. In comparison to the annealed sample, the ultimate tensile strength of the DP0 and DP9 samples improved from 557 MPa to 673 MPa (by 20%) and 738 MPa (by 32%), respectively, while the yield strength of the same samples enhanced from 340 MPa to 480 MPa (by 41%) and 720 MPa (by 112%), respectively. This enhancement is due to the accumulation of dislocations during MAC, which impede dislocation motion and strengthen the material.
5. Fractographic analysis revealed a ductile failure mode in the annealed sample, while DP0 and DP9 exhibited a mixed fracture mode. In DP9, the presence of finer dimples indicates increased strength and reduced ductility because of microstructural refinement.
6. Both DP0 and DP9 samples demonstrated superior wear resistance compared to the annealed sample. However, the DP9 sample exhibited slightly lower wear resistance than DP0, likely due to the fragmentation of martensite induced by high accumulated strain, which could act as crack initiation sites during sliding wear.
7. The annealed sample displayed significant wear debris, deep scars, and delamination features due to its poor wear resistance. In contrast, the DP samples, particularly DP0, exhibited smoother worn surfaces with reduced debris, highlighting the beneficial effects of martensite formation and grain refinement on wear resistance.

## Figures and Tables

**Table 1:** Elemental composition of medium-carbon steel AISI 1040.

Elements	C	Mn	Si	P	S	Fe
wt.%	0.40	0.72	0.28	0.020	0.018	Bal.

## Data Availability

The original contributions presented in this study are included in the article and Supplementary Materials. Further inquiries can be directed to the corresponding authors.

## Supplementary Materials

The following supporting information can be downloaded at: https://www.mdpi.com/article/10.3390/ma18133139/s1, Figure S1: (a) Vickers hardness machine and (b) Pin on disk for wear testing; Figure S2: Images of the steel samples at different thermomechanical steps in the current study. (a) AISI1040; (b) polished sample;(c) DP0;(d) DP3;(e)DP6;(f)DP9;(g) tensile sample; and (h) wear sample; Figure S3: Low magnification optical microscopy image of DP9 sample.
