# Supplementary material for: Enhancing the Mechanical Performance of Dual-Phase Steel Through Multi-Axis Compression and Inter-Critical Annealing
Source: Materials (Basel). 2025 Jul 2;18(13):3139. doi: 10.3390/ma18133139 (PMC12250940; doi:10.3390/ma18133139)
Supplement: Supplementary file 1 [file materials-18-03139-s001.zip › materials-3668945-supplementary.pdf]

Supplementary Data

Enhancing the Mechanical Performance of Dual-Phase Steel through Multi-Axis Compression and Inter-critical Annealing

Pooja Dwivedi [1,*], Aditya Kumar Padap [2], Sachin Maheshwari [3], Mohammad Faseeulla Khan [4,*], Mohammed E.Ali Mohsin [5], SK Safdar Hossain [5], Hussain Altammar [4] and Arshad Noor Siddiquee [6]

[1] Department of Mechanical Engineering, Inderprastha Engineering College, Ghaziabad 201010, Uttar Pradesh, India
[2] Department of Mechanical Engineering, Bundelkhand Institute of Engineering and Technology, Jhansi 284128, Uttar Pradesh, India; padap@bietjhs.ac.in
[3] Department of Mechanical Engineering, Netaji Subhas University of Technology, New Delhi 110078, India (Formerly Netaji Subhas Institute of Technology); ssaacchhiinn@gmail.com
[4] Department of Mechanical Engineering, College of Engineering, King Faisal University, Al-Ahsa 31982, Saudi Arabia;fmohammad@kfu.edu.sa; haltammar@kfu.edu.sa
[5] Department of Chemical Engineering, College of Engineering, King Faisal University, Al-Ahsa 31982, Saudi Arabia;maa.ali@kfu.edu.sa, snooruddin@kfu.edu.sa
[6] Department of Mechanical Engineering, Jamia Millia Islamia, New Delhi 110025, India; arshadnsiddiqui@gmail.com
* Correspondence: authors: pooja11.dwivedi@gmail.com;fmohammad@kfu.edu.sa

Supplementary Figures

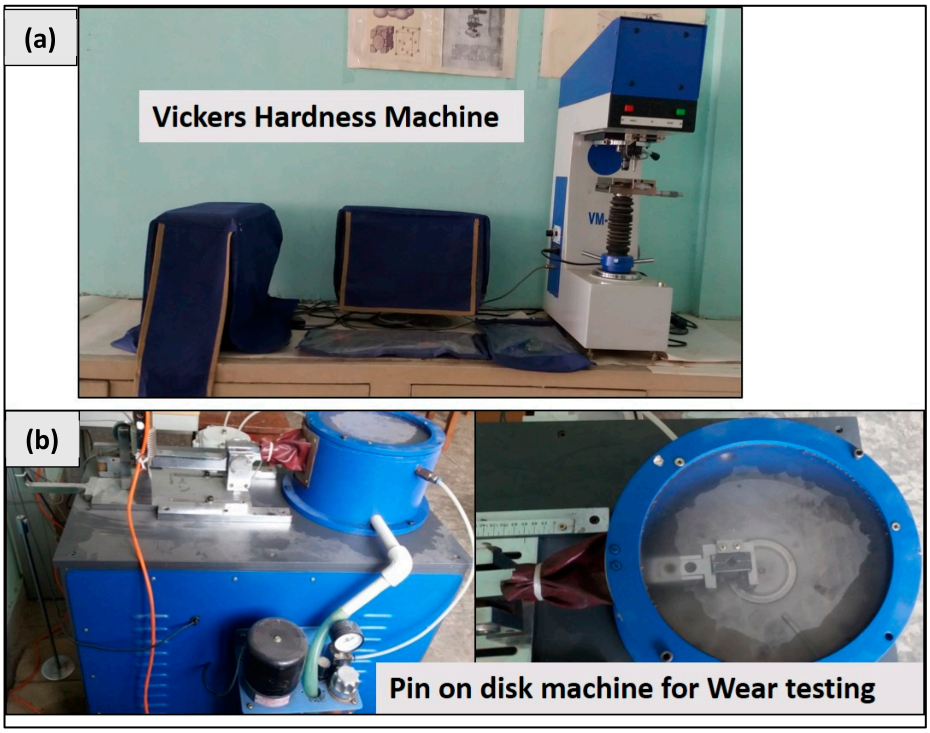

Figure S1: (a) Vickers hardness machine and (b) Pin on disk for wear testing.

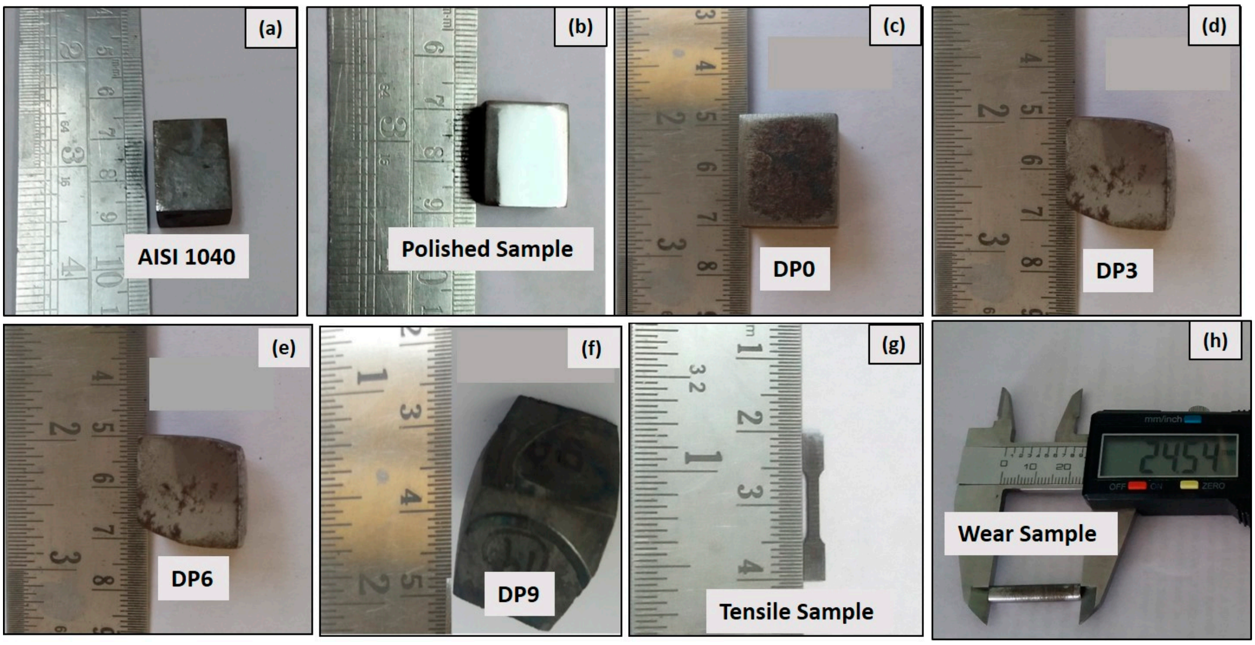

Figure S2: Images of the steel samples at different thermomechanical steps in the current study. (a) AISI1040; (b) polished sample;(c) DP0;(d) DP3;(e)DP6;(f)DP9;(g) tensile sample; and (h) wear sample.

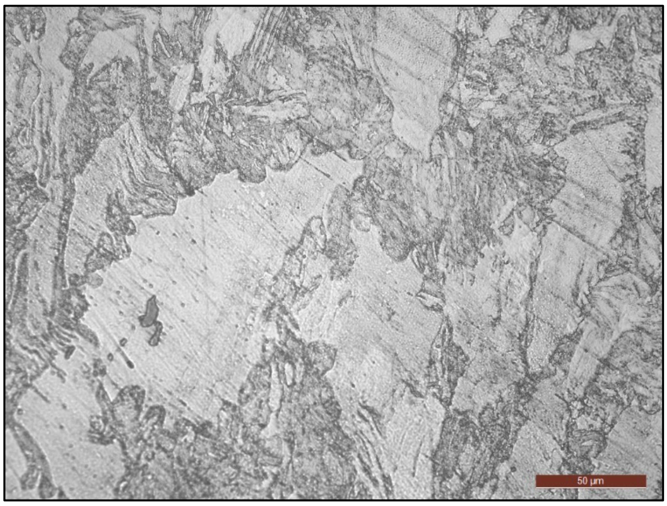

Figure S3: Low magnification optical microscopy image of DP9 sample.
